# New insights on the phylogeny, evolutionary history, and ecological adaptation mechanism in cycle‐cup oaks based on chloroplast genomes

**DOI:** 10.1002/ece3.70318

**Published:** 2024-09-17

**Authors:** Yu Li, Si‐Si Zheng, Tian‐Rui Wang, Mei‐Hua Liu, Gregor Kozlowski, Li‐Ta Yi, Yi‐Gang Song

**Affiliations:** ^1^ Eastern China Conservation Centre for Wild Endangered Plant Resources Shanghai Chenshan Botanical Garden Shanghai China; ^2^ College of Forestry and Biotechnology Zhejiang A&F University Hangzhou China; ^3^ Department of Biology and Botanic Garden University of Fribourg Fribourg Switzerland; ^4^ Natural History Museum Fribourg Fribourg Switzerland

**Keywords:** adaptive evolution, chloroplast genome, phylogenetic relationships, *Quercus* section *Cyclobalanopsis*

## Abstract

Cycle‐cup oaks (*Quercus* section *Cyclobalanopsis*) are one of the principal components of forests in the tropical and subtropical climates of East and Southeast Asia. They have experienced relatively recent increases in the diversification rate, driven by changing climates and the Himalayan orogeny. However, the evolutionary history and adaptive mechanisms at the chloroplast genome level in cycle‐cup oaks remain largely unknown. Therefore, we studied this problem by conducting chloroplast genomics on 50 of the ca. 90 species. Comparative genomics and other analyses showed that *Quercus* section *Cyclobalanopsis* had a highly conserved chloroplast genome structure. Highly divergent regions, such as the *ndhF* and *ycf1* gene regions and the *petN*—*psbM* and *rpoB—trnC‐GCA* intergenic spacer regions, provided potential molecular markers for subsequent analysis. The chloroplast phylogenomic tree indicated that *Quercus* section *Cyclobalanopsis* was not monophyletic, which mixed with the other two sections of subgenus *Cerris*. The reconstruction of ancestral aera inferred that Palaeotropics was the most likely ancestral range of *Quercus* section *Cyclobalanopsis*, and then dispersed to Sino‐Japan and Sino‐Himalaya. Positive selection analysis showed that the photosystem genes had the lowest *ω* values among the seven functional gene groups. And nine protein‐coding genes containing sites for positive selection: *ndhA*, *ndhD*, *ndhF*, *ndhH*, *rbcL*, *rpl32*, *accD*, *ycf1*, and *ycf2*. This series of analyses together revealed the phylogeny, evolutionary history, and ecological adaptation mechanism of the chloroplast genome of *Quercus* section *Cyclobalanopsis* in the long river of earth history. These chloroplast genome data provide valuable information for deep insights into phylogenetic relationships and intraspecific diversity in *Quercus*.

## Graphical abstract

Based on 50 plastomes of cycle‐cup oaks, we found a conserved structure while nine protein‐coding genes went through positive selection. The phylogenetic tree based on complete chloroplast genomes, coding DNA sequences, and highly divergent regions produced a poorly resolved genetic relationship of the plastid lineages of cycle‐cup oaks.

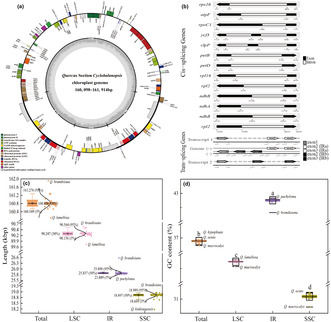

## 1 INTRODUCTION

Cycle‐cup oaks (*Quercus* section *Cyclobalanopsis*) are an important taxon of tropical and subtropical evergreen broad‐leaved forests in East and Southeast Asia (Denk et al., 2017; Denk & Grimm, 2010; Huang et al., 1999). Most cycle‐cup oaks are large trees, and only a few species living in montane cloud forests are shrubs (Li et al., 2022; Song et al., 2019; Wang et al., 2024). Morphologically, the leaf epidermal features reveal three main groups in cycle‐cup oaks that are the compound trichome base (CTB), branched uniseriate (BU), and single‐celled trichome base (STB) groups (Deng et al., 2014). Owing to the concentric circle formation of the shell bracts, cycle‐cup oaks (ca. 90 species) have been treated as a separate genus *Cyclobalanopsis* or *Quercus* subgenus *Cyclobalanopsis* for over a hundred years (Camus, 1936; Huang et al., 1999; Luo & Zhou, 2001; Menitsky, 2005; Nixon, 1993; Ørsted, 1871; Schwarz, 1936). In the recent unified systematic scheme for oaks based on morphological traits, molecular phylogenetic relationships, and evolutionary history, *Cyclobalanopsis* is downgraded to a section belonging to *Quercus* subgenus *Cerris* (Deng et al., 2018; Denk et al., 2017; Hipp et al., 2020).

With the development of molecular systematics, researchers have used genetics from different components (nDNA, cpDNA, and mtDNA) to better understand the phylogenetic relationships of *Quercus* (Li et al., 2021; Liu et al., 2022; Manos et al., 1999; McVay et al., 2017; Yang et al., 2016). However, most of these studies focus on the subgenus *Quercus*, while there are relatively few comprehensive phylogenetic studies on section *Cyclobalanopsis*. Based on restriction‐site‐associated DNA sequencing data of 35 species of *Cyclobalanopsis*, the phylogeny of cycle‐cup oaks was monophyletic and clearly divided into two main lineages (CTB and STB). Moreover, biogeographic reconstruction showed that cycle‐cup oaks originated in Palaeotropics, then spread and transferred to Sino‐Japan and Sino‐Himalaya during the Miocene (Deng et al., 2018; Hipp et al., 2020). This is the first comprehensive study on the phylogenetic and evolutionary history of section *Cyclobalanopsis* at the level of the nuclear genome inherited by both parents. However, the number of species selected in this study is still insufficient (only one‐third of the total number of species), and there is a lack of data on maternal inheritance to support the results.

Chloroplast DNA is a valid maternal genetic data for the phylogenetic analysis of *Quercus* (Petit et al., 2002; Simeone et al., 2016; Yang et al., 2017). Phylogenetic reconstruction of 147 individuals from 29 *Cyclobalanopsis* species was performed using four chloroplast DNA markers. The results showed that *Quercus* section *Cyclobalanopsis* was divided into one main clade and 11 small subclades, which were mixed with section *Ilex* (Yan et al., 2018). However, the results do not support monophyly in *Cyclobalanopsis* and the resolution is poor on some nodes. Chloroplast genomes have a typical circular quadripartite structure, generally ranging from 120 to 160 kb, and are highly conserved in terms of structure, size, and gene content (Marechal & Brisson, 2010; Palmer, 1985; Shaw et al., 2007). The whole chloroplast genome can improve the phylogenetic resolution of species to better resolve the phylogenetic relationships (Gitzendanner et al., 2018; He et al., 2021; Li, Yi et al., 2019; Tu et al., 2021; Zhai et al., 2019). Phylogenetic analysis of *Quercus* based on the whole chloroplast genomes also found that cycle‐cup oaks nested in *Quercus* section *Ilex* (Liu et al., 2021; Yang, Zhou et al., 2021; Yang, Qu et al, 2021). However, due to the minimal species coverage of cycle‐cup oaks, the phylogeny of the whole chloroplast genome in this section is not clear.

In this study, we sequenced and assembled the complete chloroplast genomes of 36 species of *Quercus* section *Cyclobalanopsis*. Additionally, 14 chloroplast genomes of *Quercus* section *Cyclobalanopsis* species were also obtained from the National Center for Biotechnology Information (NCBI) database. In total, our dataset comprised 50 of the approximate 90 species currently recognized, representing 55% of *Quercus* section *Cyclobalanopsis*. Using this dataset, we aimed to explore the structure and variations of the chloroplast genomes, reveal its phylogeny and evolution in the long river of earth history, and elucidate the adaptive mechanism of protein‐coding genes under positive selection. The findings will improve our overall understanding of the classification, phylogeny, and evolution of *Quercus* section *Cyclobalanopsis*.

## 2 MATERIALS AND METHODS

### 2.1 Taxon sampling, DNA extraction, and sequencing

Fresh and healthy plant leaves from 36 species were newly sampled from different distribution sites and desiccated in silica gel (Figure S1; Table S1). These specimens were formally identified by Dr. Yi‐Gang Song, and deposited in the Chenshan Herbarium (CSH) at Shanghai Chenshan Botanical Garden for subsequent DNA extraction. In addition, 14 chloroplast genomes of non‐duplicating species, previously published in NCBI, were also directly downloaded (Accessed on July 1 2022) (Table S1) (Cho et al., 2021; Ju et al., 2019; Li, Wang et al., 2019; Li, Wang, Liu et al.,^2020^; Li, Wang, Zhao et al.,^2020^; Li, Luo et al., 2021; Li et al., 2022; Su et al., 2019; Wang et al., 2021; Yang et al., 2018).

Total plant genomic DNA was extracted using a modified cetyl trimethyl ammonium bromide protocol (Doyle, 1987). The extracted DNA was used to construct DNA sequencing libraries with an average insert size of 350 bp, based on the whole‐genome shotgun strategy (Batzoglou et al., 1999). Paired‐end (PE) sequencing of the libraries was then performed using an Illumina NovaSeq 6000 (San Diego, CA, USA), according to the manufacturer's protocol, at Wuhan Benagen Technology Co., Ltd. (Wuhan, China). Raw data were converted using base calling analysis with reads of 150 bp in length (Ewing & Green, 1998).

### 2.2 Sequence assembly and annotation

Low‐quality reads from raw data were filtered using the SOAPnuke v1.3.0 software to obtain clean data (Chen et al., 2018). Based on clean data, the chloroplast genomes of 36 newly sequenced species in this study were de novo assembled using the GetOrganelle v1.7.6.1 software (Jin et al., 2020). All 36 newly sequenced and 14 downloaded chloroplast genomes were annotated using the online annotation tool GeSeq (https://chlorobox.mpimpgolm.mpg.de/geseq.html) (Tillich et al., 2017), and manually checked and adjusted. Chloroplast genome maps were drawn using the online program OGDRAW v1.3.1 (https://chlorobox.mpimp‐golm.mpg.de/OGDraw.html) (Greiner et al., 2019). The online program CPGView (http://47.96.249.172:16085/cpgview/home) was used to view the genetic structure of the spliced genes in the chloroplast genomes (Liu et al., 2023). Finally, the newly obtained genome sequences were uploaded and saved to the NCBI database under GenBank accession numbers OQ603420–OQ603455 (Table S1), and the raw sequence data were deposited in the Genome Sequence Archive in National Genomics Data Center, China National Center for Bioinformation/Beijing Institute of Genomics, Chinese Academy of Sciences (GSA: CRA017174) that were publicly accessible at https://ngdc.cncb.ac.cn/gsa (Chen et al., 2021; CNCB‐NGDC Members and Partners, 2022).

### 2.3 Genome structure analyses

The basic structural characteristics of the chloroplast genomes, such as genome length, GC content, and gene number, were calculated using the Geneious R9.0.2 software (Kearse et al., 2012). Simple sequence repeats (SSRs) with one to six base pairs as the basic repeat unit were identified using the MISA Perl script (https://webblast.ipk‐gatersleben.de/misa/) (Beier et al., 2017). The thresholds for repeat units of the SSRs were set to ten for mono‐; five for di‐; four for tri‐; and three for tetra‐, penta‐, and hexanucleotides. The online program Tandem Repeats Finder (TRF) v4.09 (https://tandem.bu.edu/trf/trf.html) was used to detect minisatellite sequence repeats (M) with repeat units greater than 10 bp (Benson, 1999). The alignment parameters for match, mismatch, and indels were set as two, seven, and seven, respectively, and the minimum alignment score and maximum period size were set to 80 and 500, respectively. REPuter website (https://bibiserv.cebitec.uni‐bielefeld.de/reputer) was used to predict four dispersed repeat sequences: forward (F), reverse (R), complementary (C), and palindromic (P) (Kurtz et al., 2001). Thirty and three were set as the minimum repeat size and hamming distance, respectively (Liang et al., 2019). For non‐repeating sequences and using the screening criteria of a length (≥300 bp) and the presence of both initial (ATG) and termination codons (TAA, TGA, or TAG), a total of 51 common coding sequences (CDS) were selected for codon bias analysis by the CodonW v1.4.2 software (Wright, 1990).

### 2.4 Comparative genome analyses

The boundary genes between the inverted repeat (IR) and single copy (SC) regions of each chloroplast genome were compared using the online tool IRscope (https://irscope.shinyapps.io/irapp/), which was specifically designed for the chloroplast genome (Amiryousefi et al., 2018). Fifty chloroplast genomes were aligned to assess possible structural changes, such as gene order rearrangements, inversions, and insertions, using the progressive mauve algorithm of the Mauve v2.4.0 software (Darling et al., 2004). Using the chloroplast genome of *Quercus glauca* as a reference genome, the mVISTA software (https://genome.lbl.gov/vista/mvista/submit.shtml) was used to compare interspecific variations in the 50 species at the chloroplast genome level in the Shuffle‐LAGAN mode (Brudno et al., 2003; Frazer et al., 2004). Sliding window analysis was performed using the DnaSP (DNA Sequence Polymorphism) v6.12.03 software to calculate the nucleotide variability (Pi) values and detect highly variable sites among the chloroplast genomes with step size of 200 bp and window length of 800 bp (Rozas et al., 2017).

### 2.5 Phylogenetic analyses

To infer the phylogenetic relationships of *Quercus* section *Cyclobalanopsis*, we performed phylogenetic analyses using three data sets: (1) the complete chloroplast genomes (CCGs); (2) the CDS of PCGs; and (3) the highly divergent regions detected in comparative genomic analyses (gene regions *psbC*, *ndhF*, and *ycf1*; and intergenic spacer regions *petN—psbM*, *psbZ—trnG‐GCC*, *rpl32—trnL‐UAG*, *trnK‐UUU—rps16*, *trnH‐GUG—psbA*, *rpoB—trnC‐GCA*, *trnF‐GAA—ndhJ*, and *ndhF—rpl32*). *Quercus serrata* (MK922350), *Quercus acutissima* (MH899015), and *Quercus aquifolioides* (KX911971) were chosen as outgroups to root the phylogenetic trees. Multiple sequences were aligned using the MAFFT v7.450 software with default parameters (Katoh & Standley, 2013). Maximum likelihood (ML) phylogenetic trees were constructed using the IQ‐tree v2.1.3 software with 1000 ultrafast bootstrap replicates (Minh et al., 2020). The best‐fitting models were selected using ModelFinder in IQ‐tree v2.1.3 (Kalyaanamoorthy et al., 2017). Finally, the constructed phylogenetic trees were visualized and beautified in the FigTree v.1.4.4 software (http://tree.bio.ed.ac.uk/software/figtree/).

### 2.6 Ancestral aera reconstruction

Based on Flora of China, the geographical distribution information of 50 species of *Quercus* section *Cyclobalanopsis* and outgroups were obtained in Table S2 (Huang et al., 1999). According to the floristic regions divided by Wu et al. (2011), it was divided into three biogeographical regions: (A) Sino‐Japan (including Eastern Asia subtropical areas east of the Tanaka Line); (B) Sino‐Himalaya (including the subtropical areas west of the Tanaka Line and the southeast edge of the Himalayas); and (C) Palaeotropics (including tropical regions of southern Yunnan, Guangxi, Hainan, and Indo‐China) (Deng et al., 2018; Wu et al., 2011). The statistical dispersal‐vicariance analysis (S‐DIVA) in the RASP v4.3 software was employed to reconstruct the ancestral geographical distribution of *Quercus* section *Cyclobalanopsis* (Yu et al., 2015, 2020). The analysis was performed on the phylogenetic tree constructed from CCGs with the highest support value.

### 2.7 Positive selection analyses

To explore the adaptive evolution of PCGs in the chloroplast genomes of *Quercus* section *Cyclobalanopsis*, positive selection analyses were performed using the Codeml program in the PAML v4.9j software (Yang, 2007). CDS were stripped of the termination codon and aligned using the ClustalW (Codons) plugins in the MEGA‐X software (Kumar et al., 2018; Thompson et al., 2002). An ML phylogenetic tree was generated based on the CDS of 50 chloroplast genomes using the IQ‐tree v2.1.3 software (Minh et al., 2020). Six site models (seqtype = 1; model = 0; and NSsites = 0, 1, 2, 3, 7, and 8) were used to identify the selection pressure and potential positive selection sites on the 79 common PCGs. The likelihood ratio test (LRT) was used to compare models between M1 (nearly neutral) and M2 (positive selection), M0 (one ratio) and M3 (discrete), and M7 (beta) and M8 (beta and *ω*). The chi‐square test (*χ* ^2^) was used to detect significance for the PCGs with positive selection sites, and genes with *p* < .05 were selected as positive selection genes. Finally, the posterior probability of the sites was calculated based on Bayes empirical Bayes (BEB) to evaluate the significance level of positive selection sites (*p* > 95%).

## 3 RESULTS

### 3.1 Basic structural characteristics of chloroplast genomes

**FIGURE 1:**
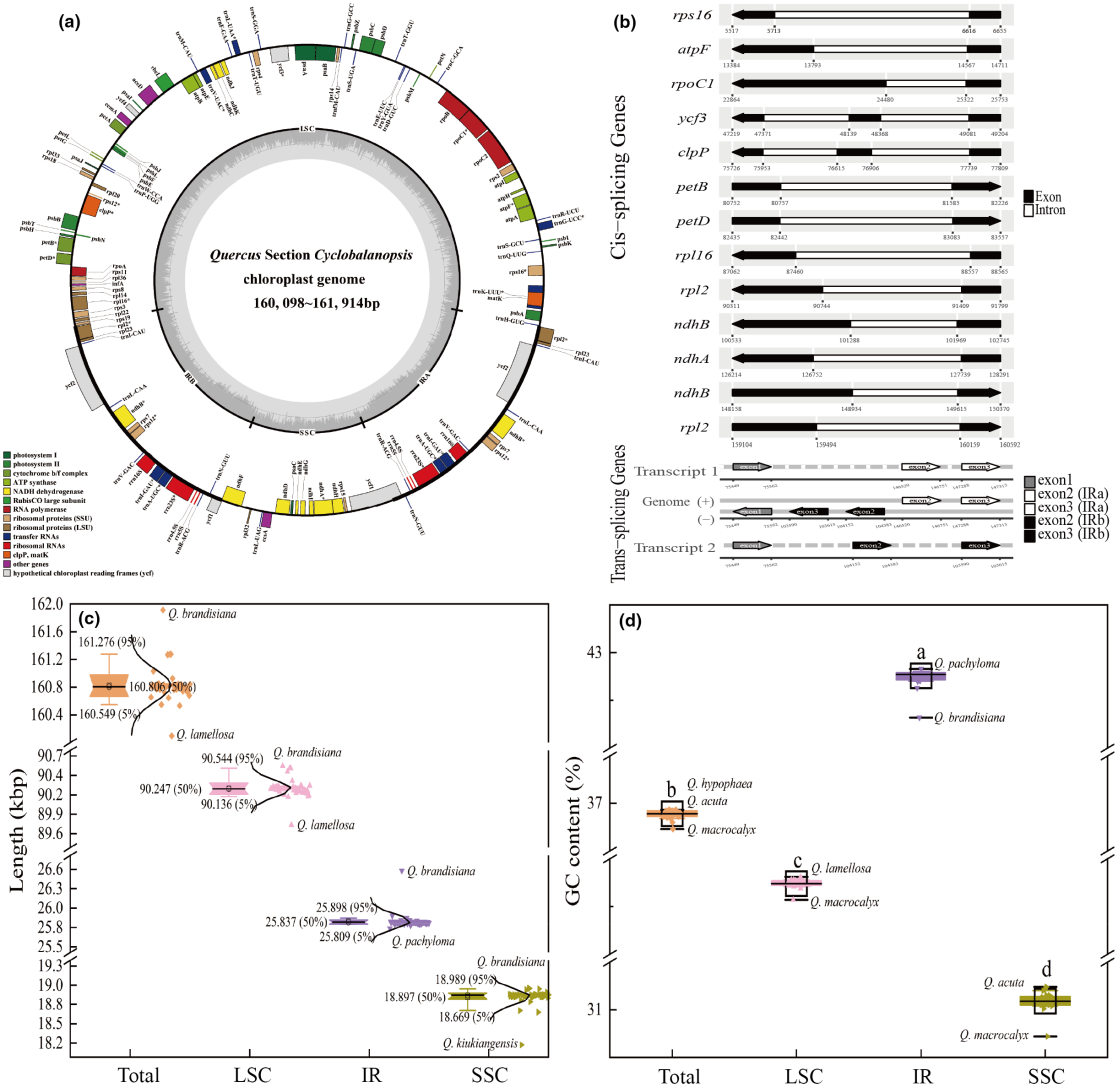
Schematic map and basic features of the chloroplast genomes of *Quercus* section *Cyclobalanopsis*. (a) Genome map of the chloroplast genomes. Genes outside the circle are transcribed in the counter‐clockwise direction, whereas those inside the circle are transcribed in the clockwise direction. The genes belonging to different functional groups are identified by different colors. The length and boundary of the LSC, SSC, and two IR regions are indicated in the inner circle. The dark gray area indicates the GC content while the lighter gray corresponds to the AT content of the genomes. IR, inverted repeat; LSC, large single copy; SSC, small single copy. (b) Gene structure map of the cis‐spliced genes and the trans‐spliced gene *rps12* in the chloroplast genomes. The cis‐spliced genes are arranged from top to bottom in the order they are found in the chloroplast genomes. The gene names are shown on the left, and the gene structures are on the right. The exons are shown in black and the introns are shown in white. The arrows indicate the direction of the genes. (c, d) The length (c) and GC content (d) of each region of the chloroplast genomes.

The length of the assembled chloroplast genomes of *Quercus* section *Cyclobalanopsis* ranged from 160,098 bp (*Quercus lamellosa*) to 161,914 bp (*Quercus brandisiana*) (Figure 1). All chloroplast genomes were highly conserved and showed a typical circular quadripartite structure comprising a large single copy (LSC) region (89,725–90,577 bp) and a small single copy (SSC) region (18,175–18,996 bp) separated by a pair of IR regions (25,748–26,581 bp) (Figure 1c; Table S3). The average GC content (GC%) varied from 36.73% to 36.93%, with that of the IR region (42.32%–42.83%) being significantly higher than that of the LSC (34.57%–34.81%) and SSC regions (30.72%–31.24%) (Figure 1d; Table S3).

The total number of annotated genes was uniform among the 50 chloroplast genomes, including 86 PCGs, 37 transfer RNA (tRNAs), and eight ribosomal RNA genes (rRNAs). Eighteen genes, comprising seven PCGs, seven tRNAs, and four rRNAs, were duplicated in the two IR regions (Figure 1a; Table 1). The *rps12* and *ycf1* genes spanned two regions between the IR and LSC/SSC, respectively (Figure 1a). The 113 unique genes were divided into four categories according to their functions, with transcription and translation, photosynthesis, biosynthesis, and unknown containing 60, 44, 5, and 4 genes, respectively. A total of 18 genes contained introns, among which 15 genes, *trnK‐UUU*, *trnG‐GCC*, *trnL‐UAA*, *trnV‐UAC*, *trnI‐GAU*, *trnA‐UGC*, *rps16*, *rpl2*, *rpl16*, *rpoC1*, *atpF*, *ndhA*, *ndhB*, *petB*, and *petD*, contained one intron, and the other three genes, *ycf3*, *clpP*, and *rps12*, contained two introns (Table 1). Of the 12 PCGs spliced by introns, 11 were cis‐spliced genes, whereas *rps12* was a trans‐spliced gene (Figure 1b).

**TABLE 1:** Genetic classification of the chloroplast genomes of *Quercus* section *Cyclobalanopsis*.

Category	Group	Name
Transcription and translation	Translational initiation factor	*infA*
	Ribosomal RNAs	*rrn16S*(×2), *rrn4.5S*(×2), *rrn23S*(×2), *rrn5S*(×2)
	Transfer RNAs	*trnR‐UCU*, *trnfM‐CAU*, *trnD‐GUC*, *trnH‐GUG*, *trnM‐CAU*, *trnE‐UUC*, *trnS‐GGA*, *trnF‐GAA*, *trnP‐UGG*, *trnT‐UGU*, *trnG‐UCC*, *trnQ‐UUG*, *trnY‐GUA*, *trnW‐CCA*, *trnS‐UGA*, *trnC‐GCA*, *trnT‐GGU*, *trnL‐UAG*, trnK‐UUU*, trnV‐UAC*, trnL‐UAA*, trnG‐GCC*, trnA‐UGC*(×2), trnI‐GAU*(×2), *trnL‐CAA*(×2), *trnI‐CAU*(×2), *trnN‐GUU*(×2), *trnV‐GAC*(×2), *trnR‐ACG*(×2)
	Small subunit of ribosome (SSU)	*rps2*, *rps11*, *rps19*, *rps14*, *rps4*, *rps15*, rps16*, *rps8*, *rps18, rps3*, *rps12*(×2)**, *rps7*(×2)
	Large subunit of ribosome (LSU)	*rpl14*, *rpl20*, *rpl36*, *rpl33*, rpl16*, *rpl32*, *rpl22*, rpl2*(×2), *rpl23*(×2)
	DNA‐dependent RNA polymerase	*rpoC2*, *rpoB*, rpoC1*, *rpoA*
Photosynthesis	Photosystem I	*psaB*, *psaJ*, *psaA*, *psaI*, *psaC*
	Photosystem II	*psbA*, *psbC*, *psbH*, *psbZ*, *psbI*, *psbJ*, *psbK*, *psbF*, *psbD*, *psbT*, *psbN*, *psbL*, *psbM*, *psbE*, *psbB*
	Subunit of cytochrome	petB*, *petN*, *petL*, *petG*, petD*, *petA*
	ATP synthase	*atpA*, *atpI*, *atpB*, *atpE*, atpF*, *atpH*
	RubisCO large subunit	*rbcL*
	NADH dehydrogenase	*ndhG*, *ndhD*, *ndhE*, *ndhK*, *ndhH*, *ndhI*, *ndhF*, ndhA*, *ndhJ*, *ndhC*, ndhB*(×2)
Biosynthesis	Maturase	*matK*
	ATP‐dependent protease	clpP**
	Acetyl‐CoA‐carboxylase	*accD*
	Envelop membrane protein	*cemA*
	C‐Type cytochrome synthesis	*ccsA*
Unknown	Hypothetical chloroplast reading frames (ycf)	*ycf4*, ycf3**, *ycf1*(×2), *ycf2*(×2)

*Note*: Genes marked with the asterisk are those with single (*) or double (**) introns. Duplicate genes located in the IR regions are marked as (×2).

### 3.2 Repeat sequences

**FIGURE 2:**
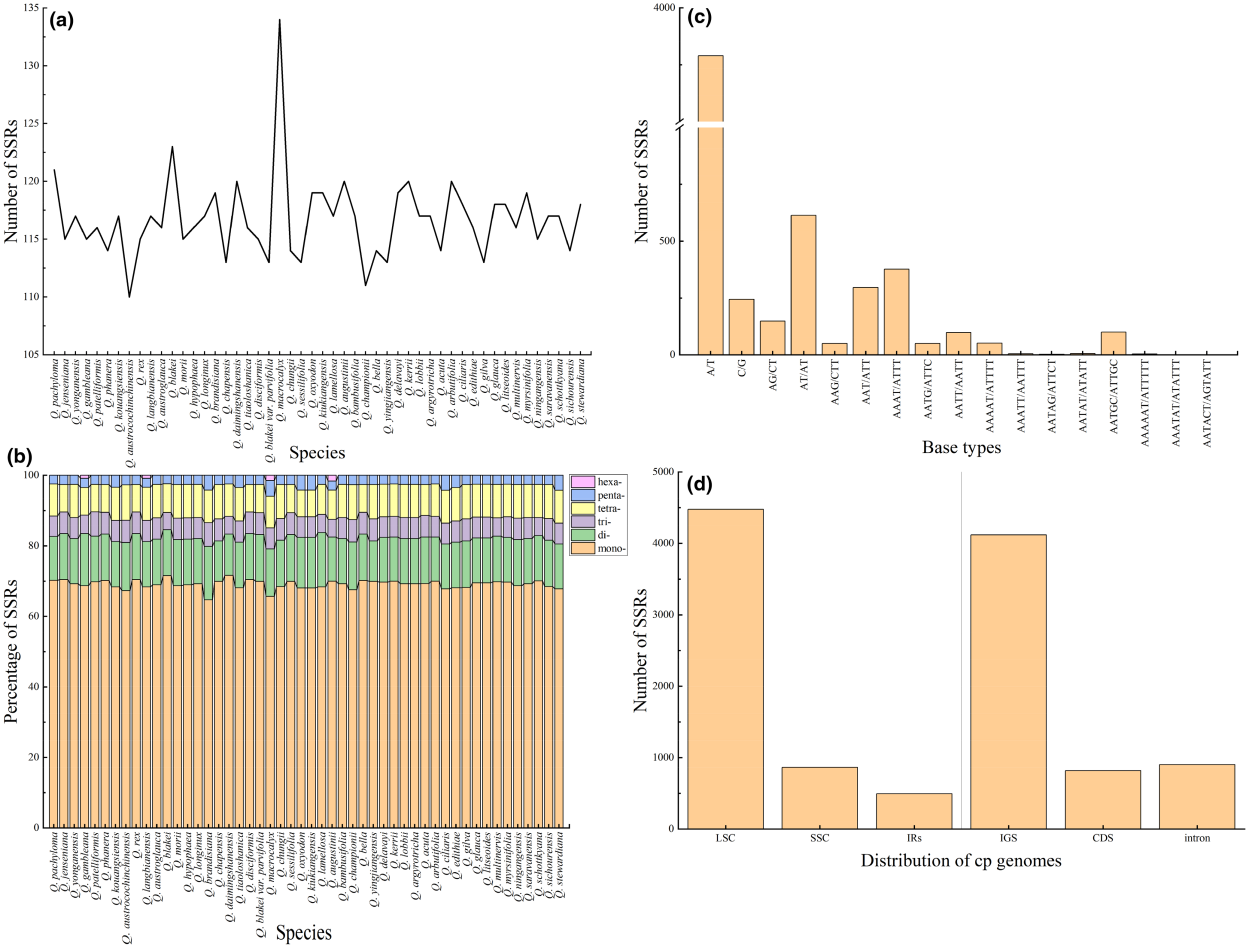
Type, distribution, and number of SSRs in 50 chloroplast genomes of *Quercus* section *Cyclobalanopsis*. (a) Total SSR numbers in each chloroplast genome. (b) The percentage of SSRs with six repeat types (mono‐, di‐, tri‐, tetra‐, penta‐, and hexanucleotide). (c) The number of SSRs with different base types. (d) The number of SSRs in different regions of the chloroplast genomes. CDS, coding sequences; IGS, intergenic spacers.

**FIGURE 3:**
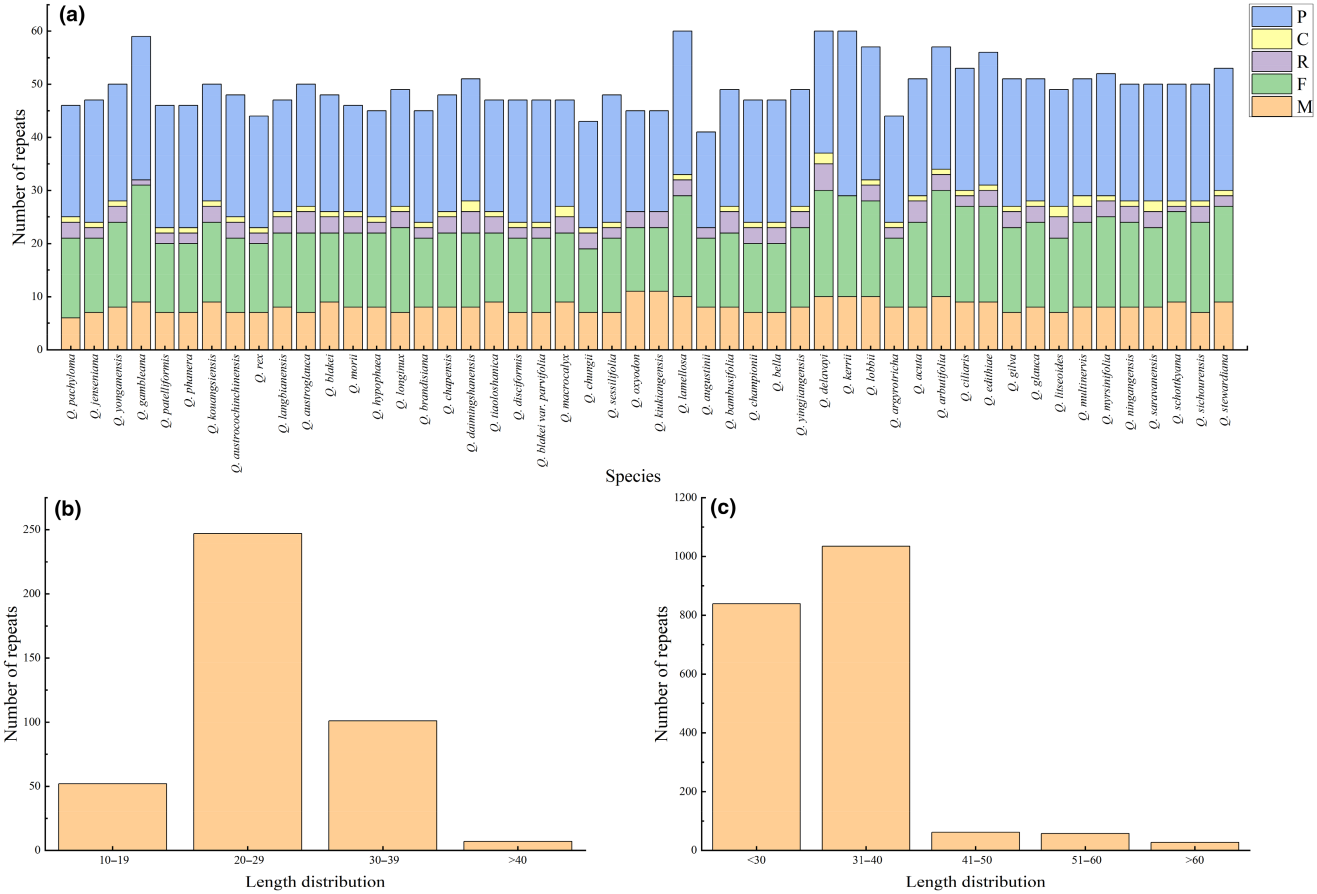
The number and length distribution of various repeat types in 50 chloroplast genomes of *Quercus* section *Cyclobalanopsis*. (a) Number of different repeat types in each chloroplast genome. C, complementary repeat sequences; F, forward repeat sequences; M, minisatellite repeat sequences; P, palindromic repeat sequences; R, reverse repeat sequences. (b, c) The length distribution of minisatellite (b) and dispersed (c) repeat sequences.

The number and distribution of SSRs, minisatellites, and dispersed repeat sequences were analyzed in the chloroplast genomes of the 50 species. SSRs were most abundant, and all types of repeats were present. *Quercus macrocalyx* contained the most SSRs (134), whereas *Quercus austrocochinchinensis* had the lowest (110) (Figure 2a; Table S4). The amount of mononucleotide repeats was the highest (74–88), accounting for approximately 70% of the total, followed by dinucleotide repeats; with slightly more tetranucleotide than trinucleotide repeats. Hexanucleotide repeats were only present in *Quercus gambleana*, *Quercus langbianensis*, *Q. macrocalyx*, and *Quercus augustinii* (Figure 2b). Most SSRs comprised two complementary bases, adenine (A) and thymine (T), indicating a strong A/T bias (Figure 2c). SSRs were distributed mainly in the LSC and intergenic spacer regions (IGS), with only a few in the SSC, IRs, intron, and coding regions (Figure 2d). A total of 407 minisatellite repeat sequences (M) were detected, ranging from six in *Quercus pachyloma* to 11 in *Quercus oxyodon* and *Quercus kiukiangensis* (Figure 3a; Table S5). The copy number of the repeat sequences was mainly between two and four, and the period size of the repeat units ranged from 11 to 101 bp and were mainly between 20 and 29 bp (Figure 3b). Among the four types of dispersed repeat sequences, palindromic (P) and forward (F) sequences were the most abundant, whereas reverse (R) and complementary (C) sequences were rare and were not detected in some species (Figure 3a; Table S5). All dispersed repeat sequences were between 30 and 120 bp in size, and most were concentrated between 30 and 40 bp (Figure 3c).

### 3.3 Codon usage bias

**FIGURE 4:**
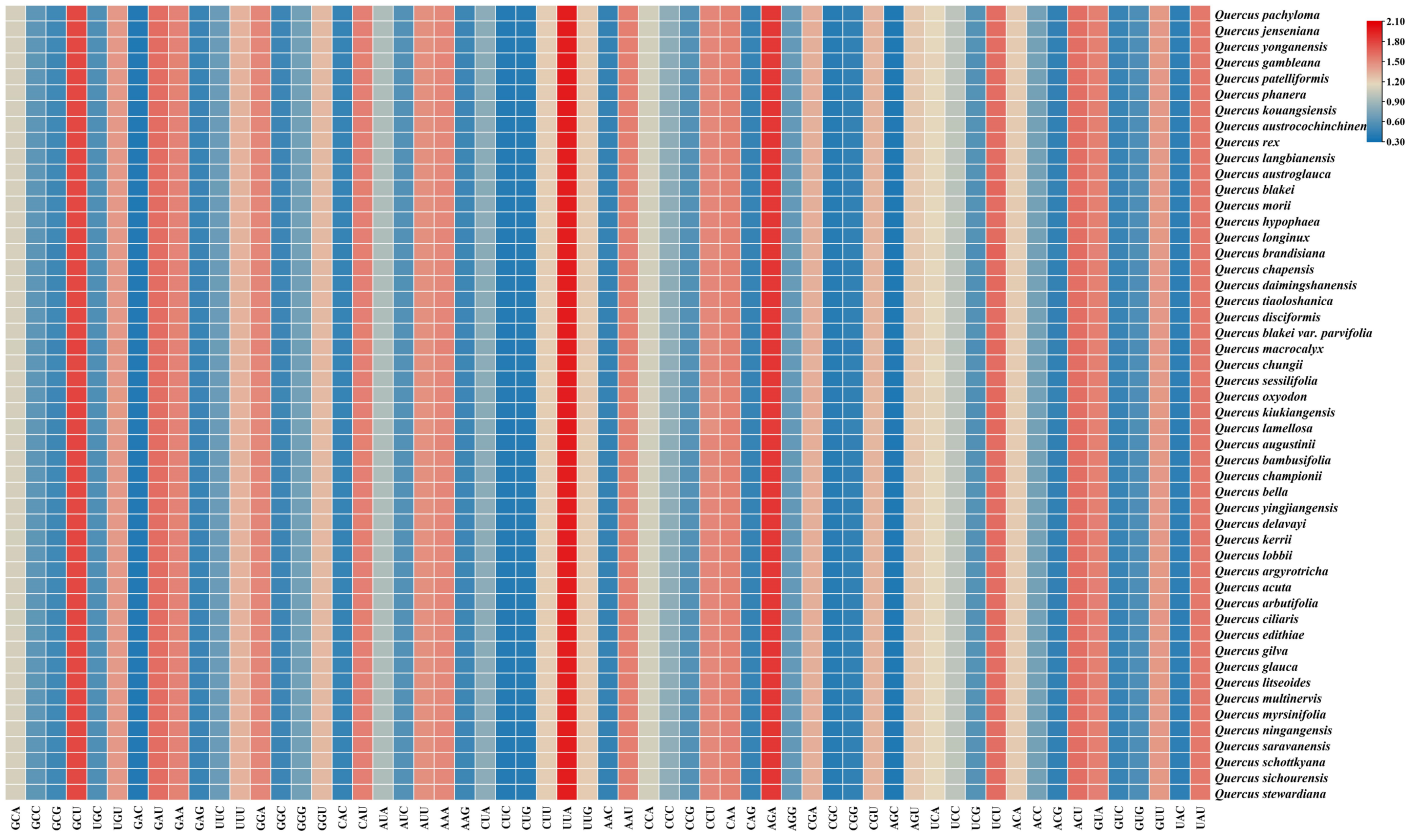
The heat map of the relative synonymous codon usage (RSCU) in 50 chloroplast genomes of *Quercus* section *Cyclobalanopsis*. In the color scale, higher red values indicate higher RSCU values and lower blue values indicate lower RSCU values.

The codon usage bias analysis of 51 selected coding sequences showed that each sequence contained 20,263 (*Q. lamellosa*) to 20,440 (*Quercus acuta*) codons, and the mean GC content of codons (GC_all) ranged from 37.72% (*Quercus rex*) to 37.89% (*Quercus morii*) (Table S6). The GC content of the first, second, and third codon sites, represented by GC1, GC2, and GC3, respectively, was less than 50% and showed a decreasing trend of GC1 > GC2 > GC3, further indicating that the chloroplast genomes were rich in A/T bases (Table S6). The effective number of codon values (ENc) were all significantly greater than 35, ranging from 49.79 (*Q. macrocalyx*) to 50.03 (*Q. lamellosa*), indicating weak codon usage bias in these chloroplast genomes (Table S6). Among the calculated relative synonymous codon usage values (RSCU) of the 59 synonymous codons, 30 codons had RSCU values greater than 1; among which only two ended in G/C (UCC and UUG), and the remaining 28 all ended in A/U with 16 ending in U and 11 ending in A. This showed that these codons have a bias in favor of the A/U endings (Table S7). The codon with the largest RSCU value was UUA, which encodes Leucine (Leu), followed by AGA, which encodes Arginine (Arg) (Figure 4).

### 3.4 Comparative genome analyses of the chloroplast genomes

**FIGURE 5:**
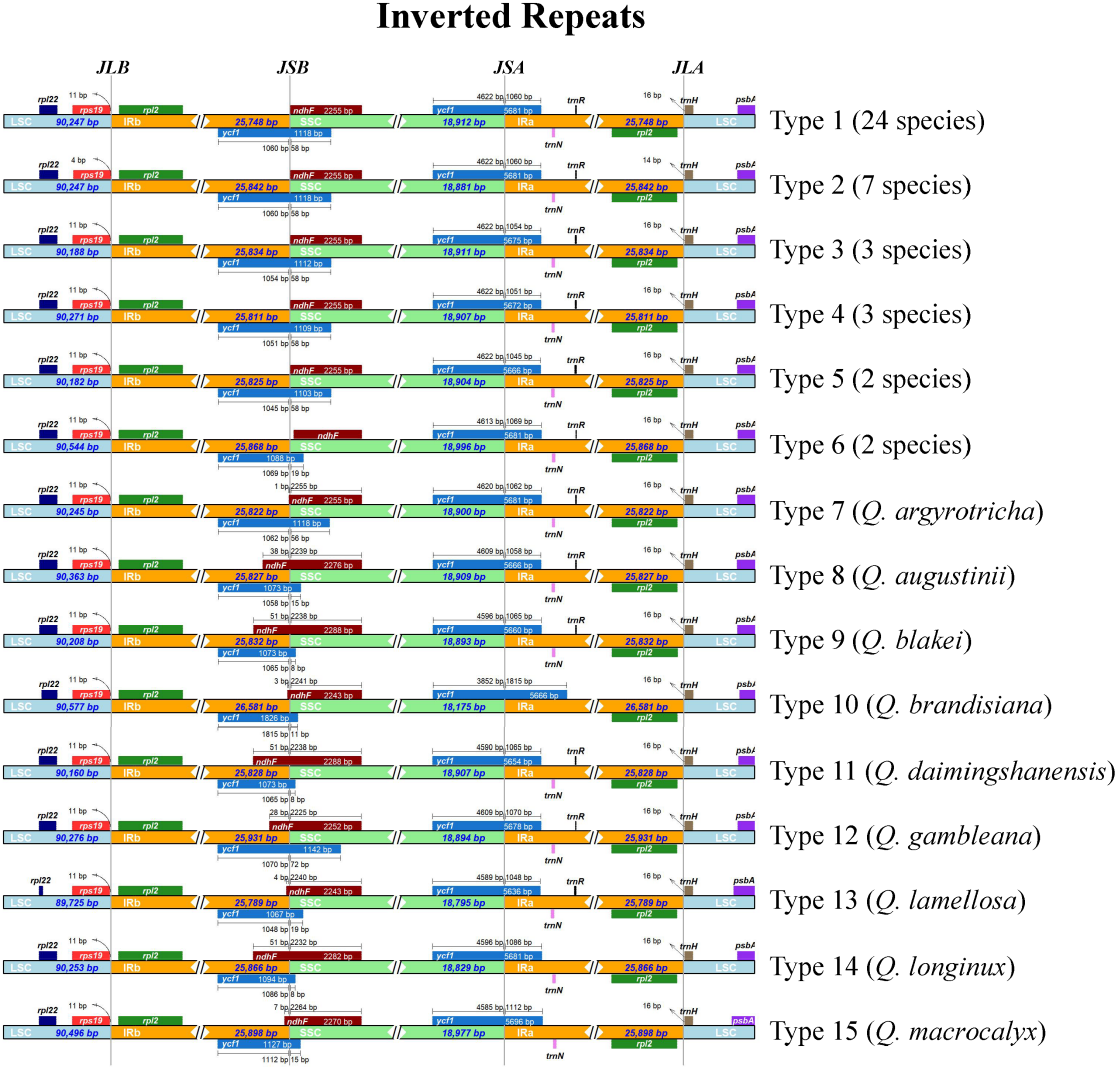
Comparison of the junction regions (JLA, JLB, JSB, and JSA) of *Quercus* section *Cyclobalanopsis*. Genes are denoted by colored boxes. The numbers above the gene boxes indicate the distance between the end of the gene and the boundary sites.

Fifteen different types of contraction and expansion of the IR regions were identified which resulted in variation in the chloroplast genomes of *Quercus* section *Cyclobalanopsis* (Figure 5; Table S8). The junction regions had the same relative position in all 50 chloroplast genomes. Both *rps19* and *trnH* were located in the LSC region and were distributed near the LSC/IRb (JLB) and LSC/IRa (JLA) boundaries, respectively. The *rps19* contracted by four bp and *trnH* expanded by 14 bp of the seven species of Type 2, whereas in the remaining species, *rps19* contracted by 11 bp and *trnH* expanded by 16 bp. The total length of *ndhF* distributed at the SSC/IRb (JSB) boundary was 2243–2288 bp, with varying degrees of expansion or contraction at the boundary of the 11 species from Type 6 to Type 15. The *ycf1* spanned the SSC and two IR regions, with the length of the repeats in the two IR regions being between 1045 and 1815 bp. The *ycf1* at the JSB boundary expanded into the SSC region from eight to 72 bp, whereas the *ycf1* at the SSC/IRa (JSA) boundary contracted into the SSC region from 3852 to 4622 bp.

Multiple alignment analysis of *Quercus* section *Cyclobalanopsis* by Mauve showed no genomic rearrangement events or inversion phenomena, indicating a good collinearity relationship (Figure S3). These results further indicate that the chloroplast genomes are highly conserved.

**FIGURE 6:**
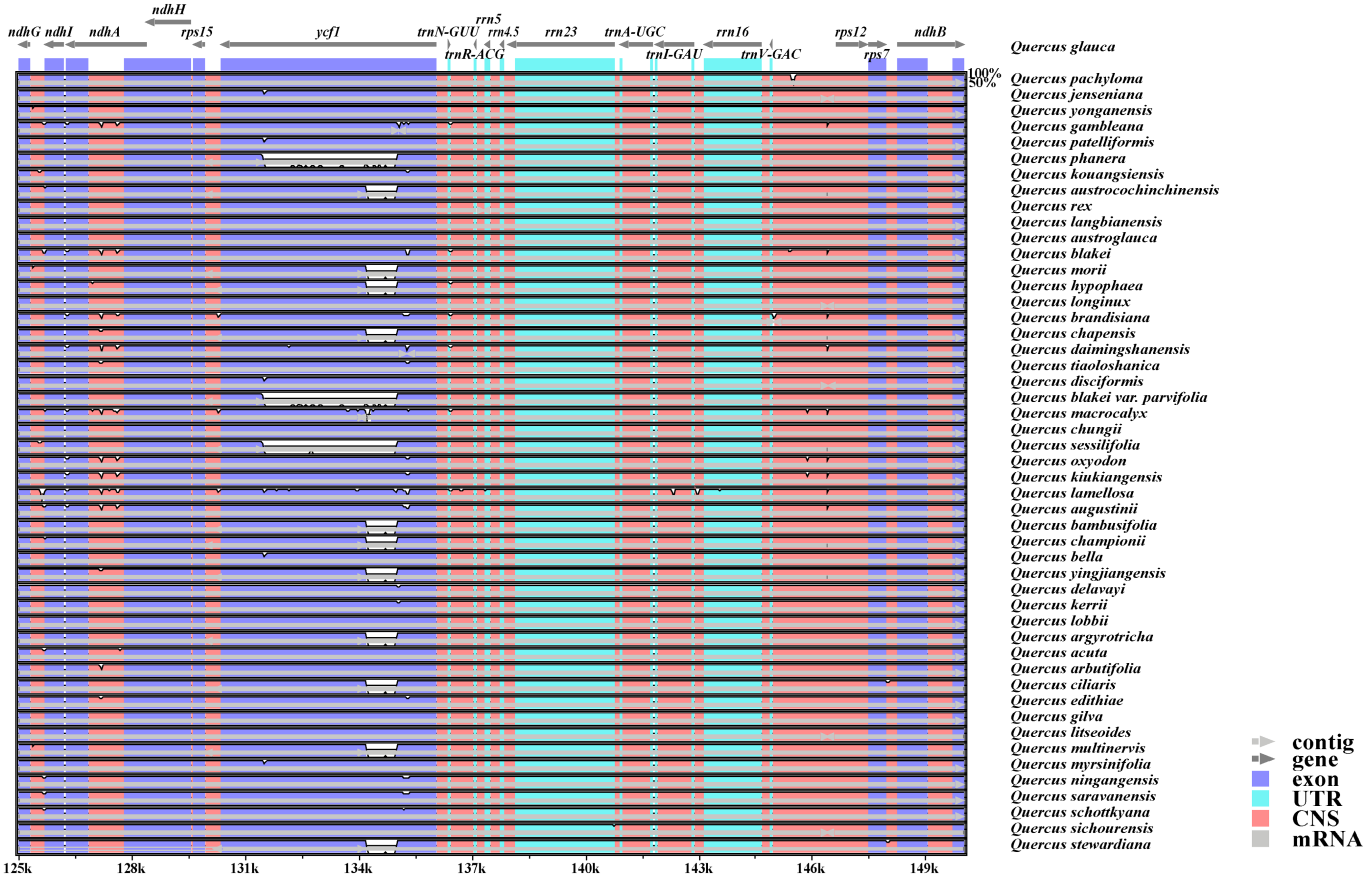
Visual local map (125–149 kb) of the alignment of *Quercus* section *Cyclobalanopsis* by the mVISTA. The gray arrows above show genes of the reference sequence, and the direction is forward or reverse. The position of the genome is shown on the horizontal axis at the bottom of each block. The alignment similarity percentages are shown on the right side of the graph (the vertical axis). Genome regions are color‐coded as exon, untranslated regions (UTR), mRNA, and conserved non‐coding sequences (CNS). The complete visualization comparison map is shown in Figure S4.

Using *Q. glauca* as the reference sequence, the mVISTA results showed that the chloroplast genomes of *Quercus* section *Cyclobalanopsis* had high sequence similarity (99.0%–99.9%). However, the noncoding and SC regions showed a higher level of divergence compared with the coding and IR regions (Figure 6 and Figure S4). High variation was observed in the exon regions of two PCGs, *ndhF*, and *ycf1*, and in the conserved noncoding regions of three intergenic spacer regions, *petN*—*psbM*, *psbZ*—*trnG‐GCC*, and *rpl32*—*trnL‐UAG*. The variation of *ycf1* was particularly significant between the chloroplast genomes (Figure 6). These divergent regions contain abundant variation information which has the potential to be used to develop molecular markers as DNA barcodes for species authentication in *Quercus* section *Cyclobalanopsis*.

**FIGURE 7:**
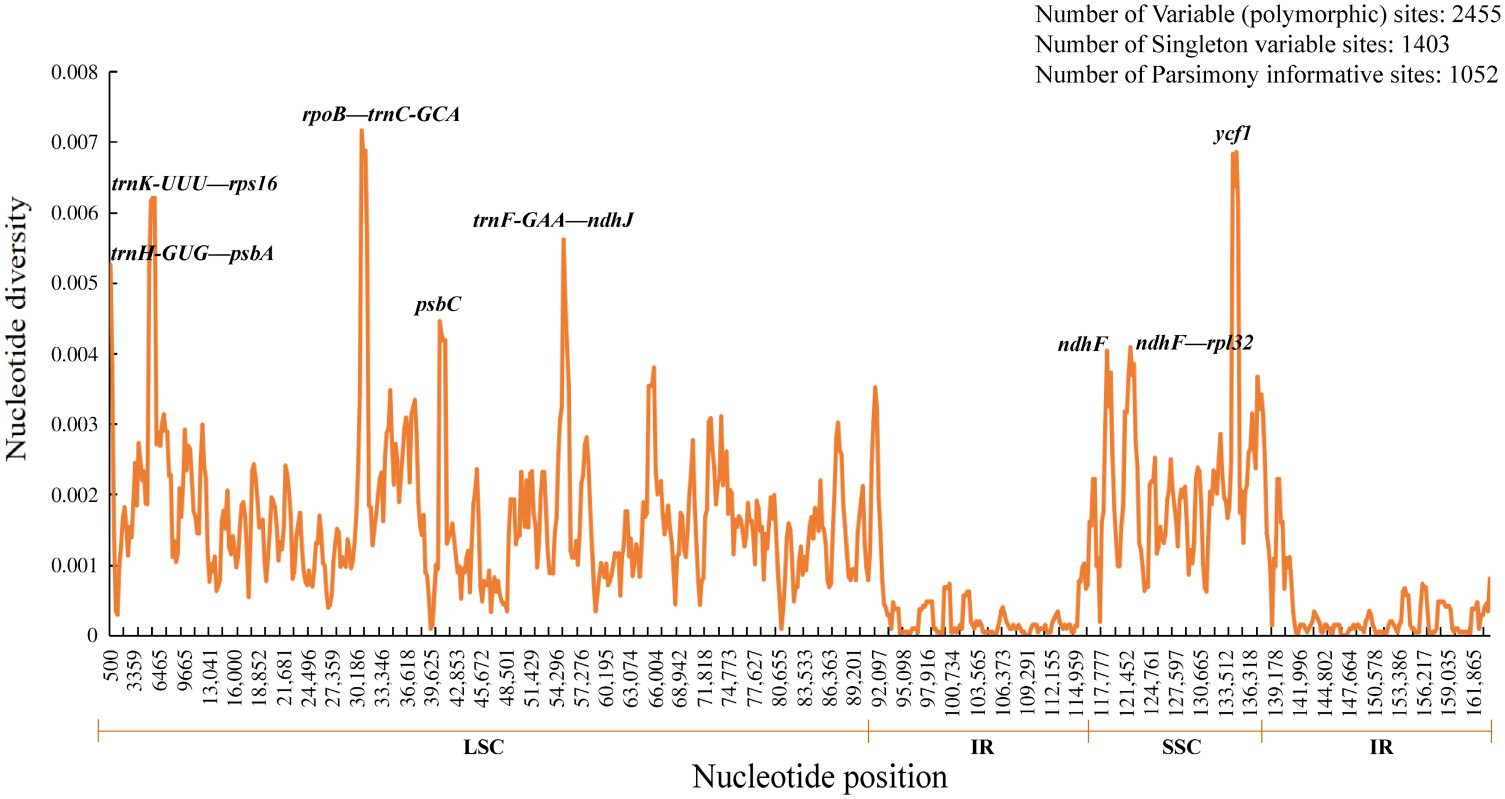
Sliding window analysis of 50 complete chloroplast genomes of *Quercus* section *Cyclobalanopsis* (window length: 600 bp; step size: 200 bp). The *X*‐axis represents nucleotide positions of the midpoint of the window and the Y‐axis represents the value of nucleotide diversity (Pi) per window.

Using DnaSP, a total of 2445 polymorphic sites were detected in the genome, including 1403 singleton variable sites (SNP) and 1052 parsimony informative sites. The range of the nucleotide diversity value was 0–0.0717 and had an average of 0.00133. The nucleotide diversity values for the IR regions (0–0.0022, average = 0.00031) were lower than those for the LSC (0–0.0717, average = 0.00173) and SSC (0–0.0686, average = 0.00225) regions. Consistent with the results of the whole‐genome comparisons, the LSC and SSC regions were much more divergent than the IR regions, and the noncoding regions also varied more than the coding regions. Furthermore, the analysis detected eight highly divergent regions (Pi > 0.004), three of which were located in the gene regions, *psbC*, *ndhF*, and *ycf1*, and five in the intergenic spacer regions, *trnK‐UUU—rps16*, *trnH‐GUG—psbA*, *rpoB—trnC‐GCA*, *trnF‐GAA—ndhJ*, and *ndhF—rpl32* (Figure 7). These highly divergent regions may have undergone rapid nucleotide substitutions during the evolution of the species, which is of great importance for phylogenetic analysis and species identification.

### 3.5 Phylogenetic relationship of *Quercus* section *Cyclobalanopsis*

**FIGURE 8:**
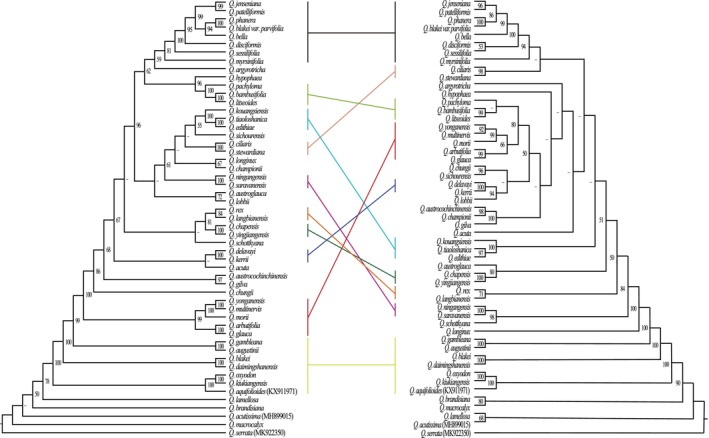
Phylogenetic tree constructed based on the CCGs and PCGs of *Quercus* section *Cyclobalanopsis* using ML methods. The bootstrap support values (BS) are labeled at the branch in the evolutionary tree, where BS less than 50% is represented by a “—.”

To infer the phylogenetic relationships within the *Quercus* section *Cyclobalanopsis*, we constructed phylogenetic trees using the ML method based on three datasets (Figure 8 and Figure S5). In all three trees, 10 (highly divergent regions), 13 (CDS), and 15 (CCGs) strongly supported clades were recognizable, and the phylogenetic relationships constructed from the CCGs data received relatively high support values. The topological structure of the three phylogenetic trees showed that *Q. acutissima* in section *Cerris* and *Q. aquifolioides* in section *Ilex* were mixed with section *Cyclobalanopsis*, and that the section *Cyclobalanopsis* was not monophyletic. The section *Cyclobalanopsis* differentiated step by step, and *Q. macrocalyx*, *Q. lamellosa*, and *Q. brandisiana* differentiated first and were located at the base of the phylogenetic trees. In the latest differentiation, section *Cyclobalanopsis* was clearly divided into two major evolutionary clades and the number of species included in this branch increased with the dataset (highly divergent regions > CDS > CCGs).

### 3.6 Ancestral aera reconstruction

**FIGURE 9:**
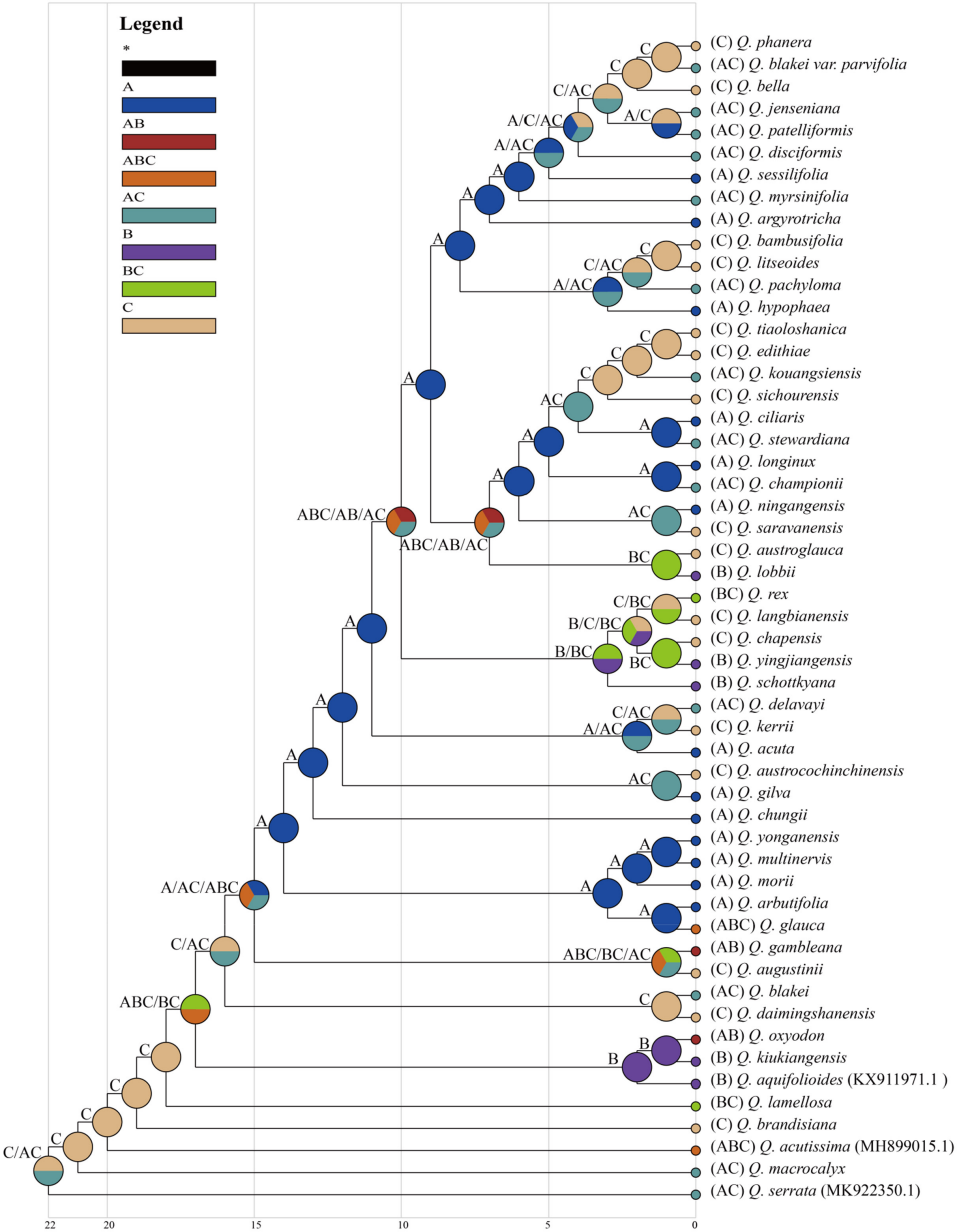
Ancestral distribution in *Quercus* section *Cyclobalanopsis* using S‐DIVA analysis based on RASP. (A) Sino‐Japan; (B) Sino‐Himalaya; and (C) Palaeotropics.

Based on the S‐DIVA analysis, we reconstructed the ancestral distribution and revealed the origin and evolutionary history of the existing *Quercus* section *Cyclobalanopsis* (Figure 9). The results showed that Palaeotropics (C) was the most likely ancestral range of the species. It then dispersed to Sino‐Japan (A) and Sino‐Himalaya (B), with Sino‐Japan (A) being the most important diffusion region. The two major evolutionary clades in the latest differentiation based on the phylogenetic tree originated together in Sino‐Japan (A) and were mainly distributed in regions Sino‐Japan (A) and Palaeotropics (C) (except *Quercus lobbii*).

### 3.7 Positive selection evolution

**FIGURE 10:**
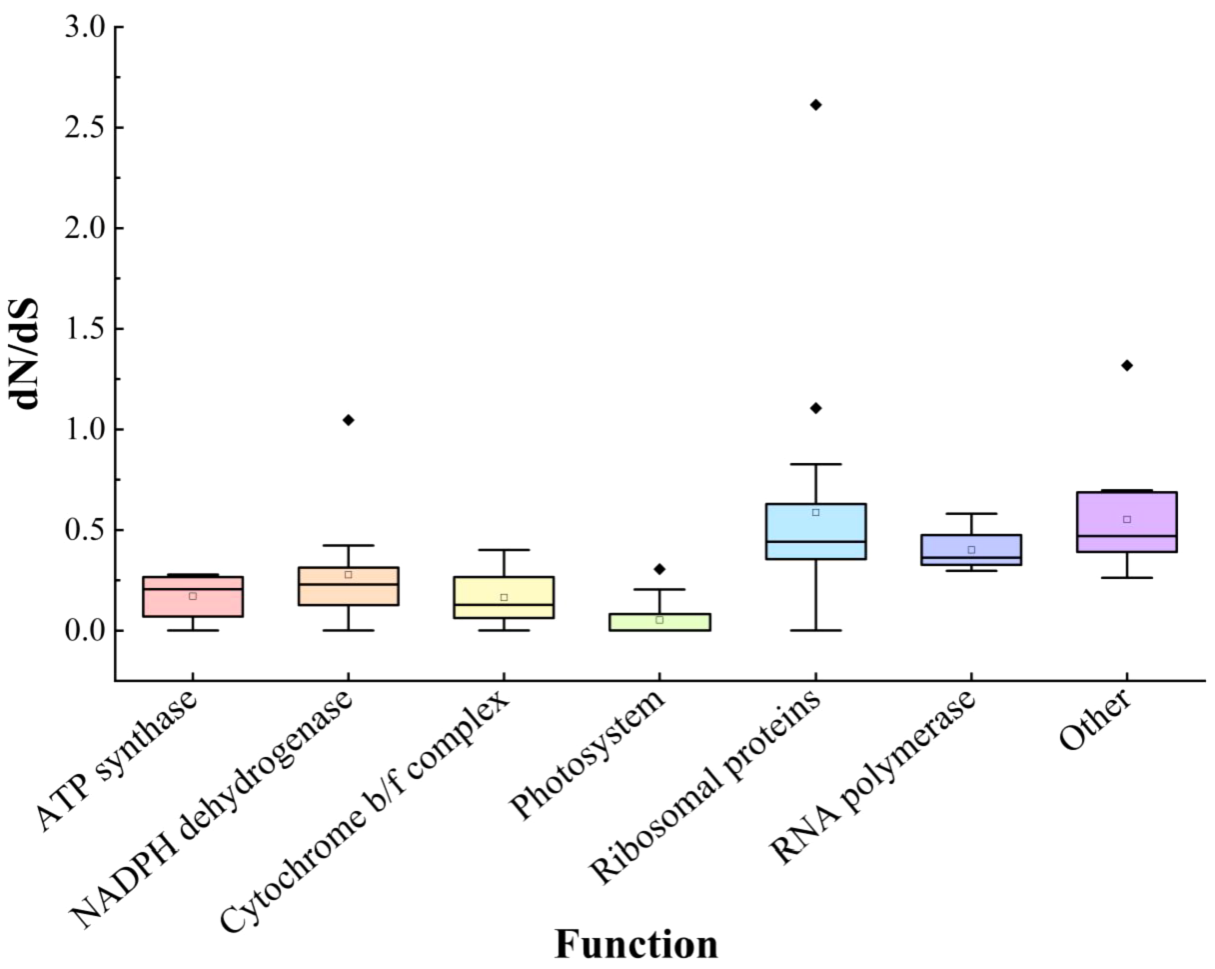
Boxplot of the values of the *ω* (dN/dS) in each functional gene group.

Seventy‐nine common PCGs were detected in the selection pressure using the PAML site‐model. The results of the model M0 (model = 0, NSsites = 0) showed that the dS value of six genes was equal to 0, which meant that the *ω* value was meaningless. The PCGs were categorized into seven functional groups based on the classification: ATP synthase, NADPH dehydrogenase, cytochrome b/f complex, photosystem, ribosomal proteins, RNA polymerase, and other genes. Among them, the photosystem genes had the lowest *ω* values, while the ribosomal protein genes had the highest *ω* values (Figure 10; Table S9).

We also identified PCGs with positive selection sites in models M2 (28) and M8 (37); rejecting the null models M1 and M7, respectively. The LRT was performed for 37 PCGs with positive selection sites based on pair‐to‐pair comparisons of M0 versus M3, M1 versus M2, and M7 versus M8. Genes with *p* < .05 were selected as positive selection genes. Nine PCGs were affected by selection pressure, including four NADH dehydrogenase genes, *ndhA*, *ndhD*, *ndhF*, and *ndhH*; RubisCO large subunit gene *rbcL*; one ribosomal protein gene *rpl32*; acetyl‐CoA‐carboxylase gene *accD*; and two hypothetical chloroplast reading frame genes, *ycf1*, and *ycf2* (Table 2). Based on BEB, there were 116 positive selection sites (M8) in these nine PCGs of which 33 had a *p* > 95% (Table 2 and Table S10). The *ycf1* gene located in the IR region harbored the highest number of positive selection sites (51), including 14 significant positive selection sites.

**TABLE 2:** Likelihood ratio test (LRT) and positive selection sites under different models of chloroplast protein‐coding genes in *Quercus* section *Cyclobalanopsis*.

Gene	Model comparison	*df*	ΔlnL	2ΔlnL	LRT (*p*‐value)	Number of positively selected sites (P > 95%)
*accD*	M0 vs. M3	4	14.456206	28.912412	8.14E‐06	
	M1 vs. M2	2	7.046054	14.092108	8.71E‐04	10 (4)
	M7 vs. M8	2	7.290798	14.581596	6.82E‐04	11 (4)
*ndhA*	M0 vs. M3	4	7.140073	14.280146	6.45E‐03	
	M1 vs. M2	2	4.464701	8.929402	1.15E‐02	5 (1)
	M7 vs. M8	2	4.469385	8.938770	1.15E‐02	7 (1)
*ndhD*	M0 vs. M3	4	15.944651	31.889302	2.02E‐06	
	M1 vs. M2	2	8.332762	16.665524	2.41E‐04	7 (1)
	M7 vs. M8	2	8.590458	17.180916	1.86E‐04	10 (2)
*ndhF*	M0 vs. M3	4	13.504137	27.008274	1.98E‐05	
	M1 vs. M2	2	4.845863	9.691726	7.86E‐03	8 (2)
	M7 vs. M8	2	5.587439	11.174878	3.74E‐03	15 (2)
*ndhH*	M0 vs. M3	4	6.127339	12.254678	1.56E‐02	
	M1 vs. M2	2	3.169165	6.338330	4.20E‐02	3 (1)
	M7 vs. M8	2	3.198039	6.396078	4.01E‐02	5 (1)
*rbcL*	M0 vs. M3	4	41.132773	82.265546	5.77E‐17	
	M1 vs. M2	2	25.996025	51.992050	5.13E‐12	4 (4)
	M7 vs. M8	2	26.239243	52.478486	4.02E‐12	4 (4)
*rpl32*	M0 vs. M3	4	9.891909	19.783818	5.50E‐04	
	M1 vs. M2	2	3.854424	7.708848	2.12E‐02	1 (1)
	M7 vs. M8	2	4.135478	8.270956	1.56E‐02	1 (1)
*ycf1*	M0 vs. M3	4	95.177045	190.354090	4.45E‐40	
	M1 vs. M2	2	50.525122	101.050244	1.14E‐22	30 (10)
	M7 vs. M8	2	51.867464	103.734928	2.98E‐23	51 (14)
*ycf2*	M0 vs. M3	4	55.836083	111.672166	3.20E‐23	
	M1 vs. M2	2	42.528727	85.057454	3.39E‐19	12 (4)
	M7 vs. M8	2	38.003763	76.007526	3.13E‐17	12 (4)

## 4 DISCUSSION

### 4.1 Chloroplast genome structure of *Quercus* section *Cyclobalanopsis*

In this study, we assembled and annotated the complete chloroplast genomes of 36 species of *Quercus* section *Cyclobalanopsis*, enriching the chloroplast genome database of section *Cyclobalanopsis* and genus *Quercus*. Similar to the chloroplast genomes of published species of section *Cyclobalanopsis* (Chen & Zhang, 2023; Li et al., 2022; Wang et al., 2021), the whole chloroplast genomes varied only slightly in size (160,098–161,914 bp) (Figure 1; Table S3) and were also highly similar in overall structure, gene order, and content (Figure 1). The GC content in the IR regions was significantly higher than that in the SC regions owing to the presence of unique rRNA genes (Yang et al., 2016; Zong et al., 2019). Compared with the chloroplast genomes of other sections of *Quercus*, the chloroplast genomes of the section *Cyclobalanopsis* were generally smaller in length, which may be one of the reasons why it has been distinguished from other *Quercus* species in the classification system (Liu et al., 2021; Pang et al., 2019; Yang et al., 2016; Yang, Zhou et al., 2021; Zhang et al., 2020).

A total of 131 genes, including 86 PCGs, 37 tRNAs, and eight rRNA genes, were annotated in the chloroplast genomes of 50 species (Table 2 and Table S3). Previous studies of the chloroplast genome sequences downloaded from the NCBI database have found that using different annotation software and reference genome sequences can lead to differences in the annotation results (Cho et al., 2021; Li, Wang, Liu et al., 2020; Yang et al., 2018). The *ycf15* gene is defined as a pseudogene because it does not encode proteins; therefore, it is not annotated in most chloroplast genomes of *Quercus*. However, it has been annotated in a few species, such as *Q. acutissima* (MH607377) and *Quercus fabri* (MK693136) (Li et al., 2018; Li, Li et al., 2021). Therefore, it is necessary to compare and verify the results of genome annotation to reduce personal errors and ensure data reliability. Gene loss and transfer to the nucleus are the major feature of chloroplast genome evolution (Kleine et al., 2009; Stegemann et al., 2003). Consistent with most angiosperms, the results showed that the chloroplast genome of *Quercus* had almost no gene deletion or transfer. However, previous studies have found that some genes, such as *ndhF*, *rpl22*, and *clpP*, have been lost or transferred during the variation and evolution of chloroplast genomes in some species (Jansen et al., 2011; Yao et al., 2015).

### 4.2 Comparison and evolution of chloroplast genomes of *Quercus* section *Cyclobalanopsis*

Repeat sequences exist widely in plant genomes and may have played an important role in plant evolution (Timme et al., 2007; Weng et al., 2014). The repeat sequences detected in this study were composed of A and T bases and had a strong A/T bias (Figure 2), which is consistent with the results of other studies (Morton, 1997; Zhang et al., 2020). The length, quantity, and distribution of various repeat sequences were highly consistent and conserved, and these differences may be caused by the size and variation of each chloroplast genome (Wu et al., 2018). Different types of repeat sequences were detected in two gene regions, *rpl12* and *ycf2*, which are likely involved in chloroplast genome rearrangement and sequence differentiation (Guisinger et al., 2010).

Codon usage bias is an important evolutionary characteristic that is common in many organisms. It is influenced by natural selection, base mutations, and other factors (Morton, 1998), and has been extensively studied in many plant species (Qin et al., 2013; Shi et al., 2022; Wang et al., 2018; Wen et al., 2021). In this study, the GC content decreased in the following order: GC1 > GC2 > GC3 (Table S6). GC content is a major factor in the formation of codon usage bias and may play an important role in the evolution of the genome structure (Yang et al., 2018). Among the 30 preferential synonymous codons, most of them terminated in A/T, except that UUG ended with G, and UCC ended with C (Figure 4). This demonstrated that the codon usages of the chloroplast genomes were biased towards A/T at the third position of codons, which was similar to reported results of other angiosperm, including the *Bupleurum*, *Chamaesium*, *Polystachya*, and *Allium* section *Bromatorrhiza* species (Chen et al., 2022; Guo et al., 2020; Jiang et al., 2022; Li, Xie et al., 2020).

The IR region plays an important role in stabilizing the chloroplast genome structure, and its expansion and contraction are the main causes of chloroplast genome length variation (Marechal & Brisson, 2010). In general, the distribution of boundary genes in *Quercus* section *Cyclobalanopsis* in this study did not show significant differences. However, the distribution of *ndhF* showed the greatest difference, being completely located in the SSC region in 39 species, whereas the remaining species had varying degrees of expansion or contraction at the JSB boundary (Figure 5). The same situation was observed in previous studies on *Punica granatum*, *Allium*, and other plants (Xie et al., 2020; Yan, Zhao et al.,^2019^). Chloroplast genomes have sequence homology; therefore, they have no gene rearrangement or inversion in most plant species (Li, Sylvester et al., 2019). In the present study, the chloroplast genome of *Quercus* section *Cyclobalanopsis* also showed good collinearity (Figure S3). Further comparison of the chloroplast genomes of *Quercus* section *Cyclobalanopsis* showed that the SC regions had a higher level of sequence diversity than the IR regions, which may be because the IR regions contained more conserved rRNA genes. This result was similar to that of other chloroplast genomes, such as *Q. acutissima*, *Lonicera japonica*, and Aceraceae (Han et al., 2016; He et al., 2017; Zhang et al., 2020). In addition, we identified highly divergent coding regions, *psbC*, *ndhF*, and *ycf1*, and noncoding regions, *petN—psbM*, *trnK‐UUU—rps16*, and *rpoB—trnC‐GCA* (Figures 6 and 7). These highly divergent regions are useful molecular resources for potential DNA barcoding and for subsequent population genetics studies of different species.

### 4.3 Phylogeny and evolutionary history of chloroplast genomes of *Quercus* section *Cyclobalanopsis*

Each angiosperm species contains three types of genomes, nuclear, chloroplast, and mitochondrial, all three of which have been used in phylogenetic analysis. Phylogenetic studies of *Quercus* are faced with great challenges, not only because of serious hybridization and introgression but also because of the inconsistent phylogenetic relationships constructed by different markers (Denk & Grimm, 2010; Manos et al., 1999; Simeone et al., 2013). In the present study, phylogenetic trees of *Quercus* section *Cyclobalanopsis* were constructed based on CCGs, PCGs, and highly divergent regions (Figure 8 and Figure S5). We also reconstructed the ancestral geographical distribution of *Quercus* section *Cyclobalanopsis* using the RASP (Figure 9).

First, some species had poor resolution between differentiation, which may be due to the low substitution rate and low genetic diversity of chloroplast genomes (Drouin et al., 2008; Yan et al., 2018; Yan, Liu, Li et al., 2019). Previous studies had shown that the species resolution of chloroplast markers in East Asian oak was poor (Ohyama et al., 2001; Yang et al., 2017), and the genetic diversity of section *Cyclobalanopsis* was the lowest in the subgenus *Cerris* (Yan et al., 2018; Yan,Liu, Li et al., 2019), suggesting that the chloroplast genome may not be able to infer a systematic classification of closely related species of *Quercus* section *Cyclobalanopsis* (Pham et al., 2017). Secondly, the species of *Quercus* section *Cyclobalanopsis* differentiated one by one during the evolutionary process and were obviously divided into two major evolutionary clades in the latest differentiation. This differed from the phylogenetic relationships previously constructed from the restriction‐site‐associated DNA sequencing data of 35 species of *Quercus* section *Cyclobalanopsis* which were divided into two main clades, named STB and CTB, respectively (Deng et al., 2018; Hipp et al., 2020). The species of each clade based on the chloroplast and nuclear genome were not consistent owing to hybridization and introgression. Maternally inherited chloroplast DNA was easily transferred during hybridization (Dumolin et al., 1995). In the reconstruction of ancestral distribution, it was revealed that the most probable ancestral distribution area of the existing species of *Quercus* section *Cyclobalanopsis* was Palaeotropics (C), and then dispersed to Sino‐Japan (A) and Sino‐Himalaya (B) (Figure 9). This result was consistent with the reconstruction of the ancestral region based on the nuclear genome (restriction‐site‐associated DNA) (Deng et al., 2018). However, the reconstruction of ancestral distribution based on the chloroplast genome of *Quercus* section *Cyclobalanopsis* showed a strong geographical structure. Therefore, the phylogeny of cpDNA can only reflect the maternal evolutionary history (Yan et al., 2018). Finally, *Quercus* section *Cyclobalanopsis* did not form a monophyletic group in our analysis. Species of the section *Cyclobalanopsis* were mixed with those from two other sections of the subgenus *Cerris*, which was consistent with the results of previous studies (Yan et al., 2018). In a basic morphological study, *Quercus* section *Cyclobalanopsis* was closely related to the section *Cerris* and also provided evidence for their nesting (Deng et al., 2014). The non‐monophyly of the chloroplast genomes and the close relationships among the sections may reflect ancient introgression or incomplete lineage sorting of the chloroplast genome in the ancestor lineages of subgenus *Cerris* (Yan et al., 2018; Yan, Liu, Li et al., 2019). In summary, the phylogenetic relationship of *Quercus* is complex and needs further analysis from multiple perspectives.

### 4.4 Adaptive evolution and mechanisms of chloroplast genomes of *Quercus* section *Cyclobalanopsis*

It is generally believed that PCGs in the organelle genomes of most plants have a lower substitution rate than those in the nuclear genomes, resulting in purifying selection (Xiang et al., 2023). The substitution rate of photosystem functional genes is the smallest in the chloroplast genome (Figure 10) (Zhang et al., 2024), suggesting that it is under stronger functional constraints (Wu et al., 2017; Yan, Liu, Yuan et al., 2019), whereas ribosomal protein genes and other functional genes have large substitution rates, possibly because they have duplicated genes in IR regions that may lead to evolution at a faster rate.

However, individual genes in the chloroplast genomes still undergo positive selection, particularly those involved in photosynthesis and other metabolic pathways (Silva et al., 2023). Species of *Quercus* section *Cyclobalanopsis* are widely distributed in the tropical and subtropical regions of East Asia (Huang et al., 1999). Over a long evolutionary period, they have adapted to diverse ecological environments and occupied different ecological niches (Jin et al., 2023). At the chloroplast genome level, nine PCGs with positive selection sites were identified in the *Quercus* section *Cyclobalanopsis* (Table 2). Most of these genes are involved in photosynthesis and other metabolic pathways, validating the previous statement. Four of these were NADH dehydrogenase genes, *ndhA*, *ndhD*, *ndhF*, and *ndhH*, which are involved in photosynthesis. The NADH complex in higher plants not only promotes ATP synthesis but also participates in chloroplast protection against photooxidative stress (Li et al., 2004; Martin et al., 1996). All species of *Quercus* section *Cyclobalanopsis* are evergreen trees with a large growth altitude span, therefore, adaptation to high ultraviolet radiation intensity may be one of the important genetic bases for the adaptive evolution of the chloroplast genome. The *accD* codes for a key enzyme involved in fatty acid biosynthesis (Slabas & Fawcett, 1992) and has undergone adaptive evolution, promoting the growth and development of *Quercus* section *Cyclobalanopsis*. Adaptive evolution of this gene has also been reported in other genera (Lee et al., 2007; Zhu et al., 2016). The *rbcL* has also been subject to positive selection in many species (Azarin et al., 2021; Kapralov & Filatov, 2007). The *ycf1* and *ycf2* are the two largest hypothetical chloroplast reading frame genes, but their functions are still unknown and their evolutionary significance remains to be studied.

## 5 CONCLUSION

In this study, we newly assembled and annotated the complete chloroplast genomes of 36 species of *Quercus* section *Cyclobalanopsis*. Combined with the existing complete chloroplast genomes of 14 other species, we carried out genome structure analysis, comparative genome analysis, phylogenetic analysis, ancestral aera reconstruction analysis, and selection pressure analysis for these 50 species of *Quercus* section *Cyclobalanopsis*. These results explored the diversity structure, phylogenetic relationship, evolutionary history, and adaptative evolutionary mechanism of the chloroplast genome of *Quercus* section *Cyclobalanopsis*. In conclusion, this study provides a wealth of valuable information that will help us to harvest deeper insights into the evolutionary history, phylogeny, and taxonomy of *Quercus*.

## AUTHOR CONTRIBUTIONS

**Yu Li:** Data curation (equal); formal analysis (equal); methodology (equal); software (equal); validation (equal); visualization (equal); writing – original draft (equal). **Si‐Si Zheng:** Data curation (equal); resources (equal); visualization (equal); writing – review and editing (equal). **Tian‐Rui Wang:** Formal analysis (equal); software (equal); writing – review and editing (equal). **Mei‐Hua Liu:** Investigation (equal); supervision (equal); writing – review and editing (equal). **Gregor Kozlowski:** Project administration (equal); writing – review and editing (equal). **Li‐Ta Yi:** Conceptualization (equal); supervision (equal); writing – review and editing (equal). **Yi‐Gang Song:** Conceptualization (equal); funding acquisition (equal); project administration (equal); resources (equal); supervision (equal); writing – review and editing (equal).

## FUNDING INFORMATION

This work was supported by the National Natural Science Foundation of China, grant number 31901217, and the Special Fund for Scientific Research of Shanghai Landscaping & City Appearance Administrative Bureau, grant number G192422 and G222404.

## CONFLICT OF INTEREST STATEMENT

The authors declare that they have no conflict of interest. No approval/permission is required for the sample collection in accordance with relevant regulations and standards.

## Supporting information


Figure S1.–S5.

Table S1.–S10.

## Data Availability

The data supporting the findings of this study are openly available in the NCBI GenBank repository at https://www.ncbi.nlm.nih.gov, reference number OQ603420–OQ60345.
