# Supplementary material for: New insights on the phylogeny, evolutionary history, and ecological adaptation mechanism in cycle‐cup oaks based on chloroplast genomes
Source: Ecol Evol. 2024 Sep 17;14(9):e70318. doi: 10.1002/ece3.70318 (PMC11407850; doi:10.1002/ece3.70318)
Supplement: Supplementary file 1 — Figure S1.–S5. [file ECE3-14-e70318-s001.docx]

**
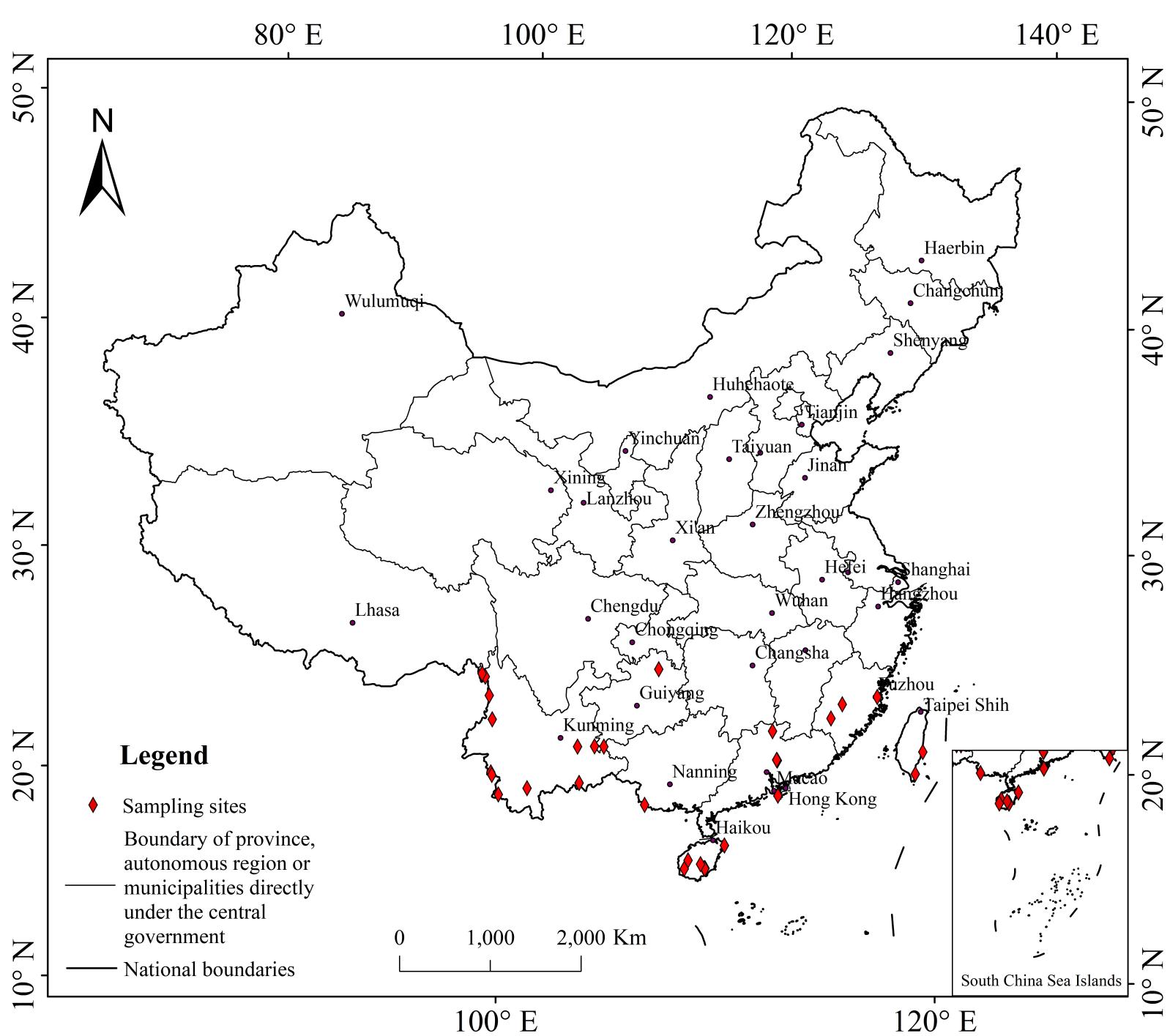
**

**Fig. S1** Sampling sites of 36 species of *Quercus* section *Cyclobalanopsis*.

**

**

**Fig. S2** The GC content at the first, second and third codon sites (GC1, GC2, and GC3) of 50 chloroplast genomes of *Quercus* section *Cyclobalanopsis*.


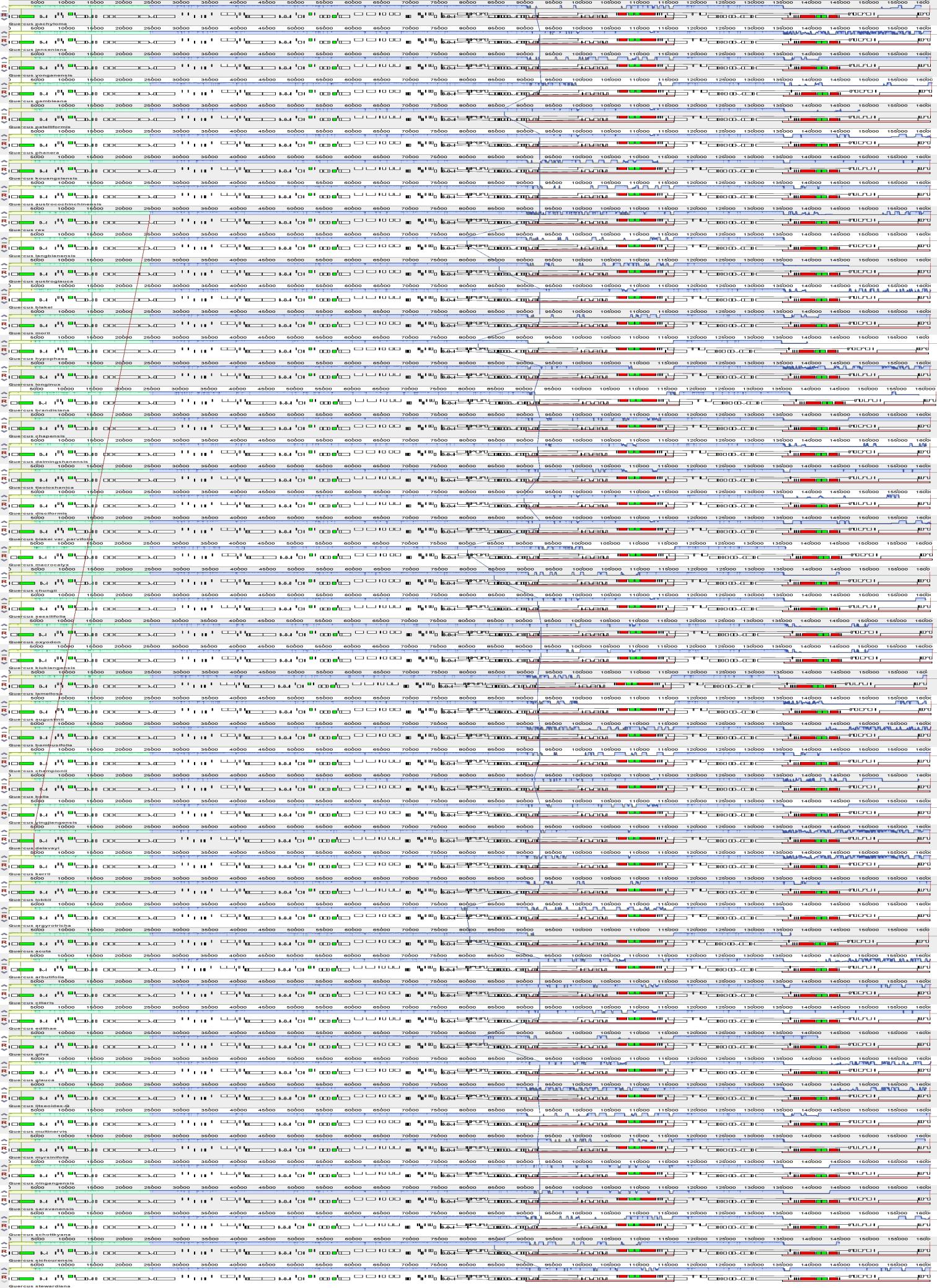


**Fig. S3** MAUVE alignment of 50 chloroplast genomes of *Quercus* section *Cyclobalanopsis*. The different color blocks represent different locally collinear blocks (LCBs). The box structure under the genome represents the corresponding annotation information: the white rectangle represents protein-coding genes, the red rectangle represents rRNA genes, and the green rectangle represents tRNA genes. The introns are connected by line segments.

**
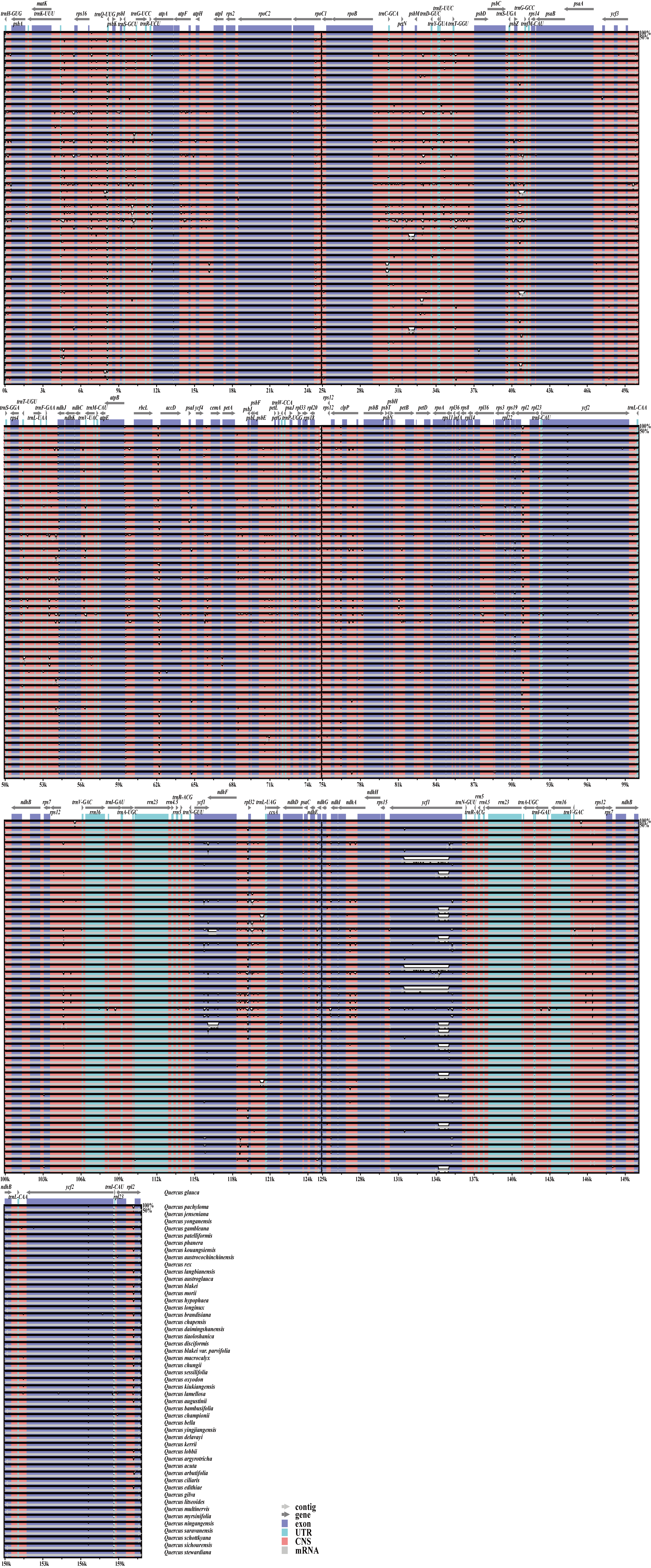
**

**Fig. S4** The complete visualization comparison map of the 50 chloroplast genomes of *Quercus* section *Cyclobalanopsis* using mVISTA.


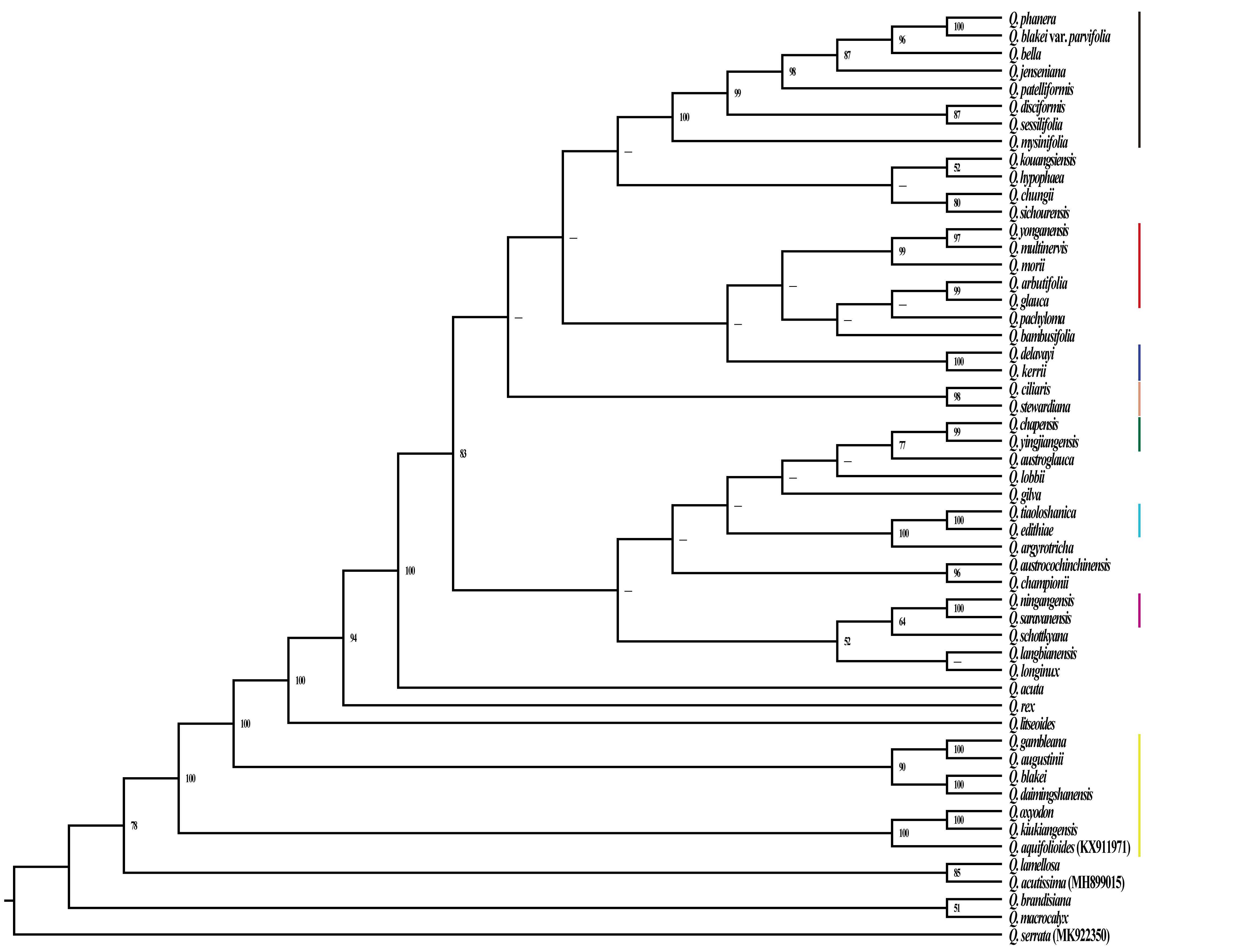


**Fig. S5** Phylogenetic tree reconstructed based on the highly divergent regions detected in comparative genomic analyses using maximum likelihood (ML) methods. The bootstrap support values (BS) are labeled at branch in the evolutionary tree, where BS less than 50 % is represented by a “—”.
